# Stem cells: a potential treatment option for kidney diseases

**DOI:** 10.1186/s13287-020-01751-2

**Published:** 2020-06-25

**Authors:** Dongwei Liu, Fei Cheng, Shaokang Pan, Zhangsuo Liu

**Affiliations:** 1 Department of Nephrology, The First Affiliated Hospital of Zhengzhou University, Zhengzhou, 450052 People’s Republic of China; 2 Research Institute of Nephrology, Zhengzhou University, Zhengzhou, 450052 People’s Republic of China; 3 Key Laboratory of Precision Diagnosis and Treatment for Chronic Kidney Disease in Henan Province, Zhengzhou, 450052 People’s Republic of China; 4 Core Unit of National Clinical Medical Research Center of Kidney Disease, Zhengzhou, 450052 People’s Republic of China

**Keywords:** Stem cells, Kidney diseases, Kidney regeneration, Organoids, Embryonic stem cells, Mesenchymal stem cells, Induced pluripotent stem cells, Progenitor cells

## Abstract

The prevalence of kidney diseases is emerging as a public health problem. Stem cells (SCs), currently considered as a promising tool for therapeutic application, have aroused considerable interest and expectations. With self-renewal capabilities and great potential for proliferation and differentiation, stem cell therapy opens new avenues for the development of renal function and structural repair in kidney diseases. Mounting evidence suggests that stem cells exert a therapeutic effect mainly by replacing damaged tissues and paracrine pathways. The benefits of various types of SCs in acute kidney disease and chronic kidney disease have been demonstrated in preclinical studies, and preliminary results of clinical trials present its safety and tolerability. This review will focus on the stem cell-based therapy approaches for the treatment of kidney diseases, including various cell sources used, possible mechanisms involved, and outcomes that are generated so far, along with prospects and challenges in clinical application.

## Introduction

Kidney diseases have become a global public health problem due to their rapidly growing incidence. These diseases affect over 10% of the global population, because of a global rise in the aging population, as well as increase in the frequency of their main etiologies, such as diabetes, cardiovascular diseases, and hypertension [1, 2]. As a routine treatment for kidney diseases, multidrug therapy cannot reverse the process of entering into end-stage renal disease (ESRD) in most of the patients, and those with ESRD require renal replacement therapy, i.e., maintenance dialysis or kidney transplantation [3]. Owing to high medical costs and adverse impacts on the patient’s quality of life, dialysis is not considered as an ideal treatment strategy [4, 5]. Kidney transplantation enables patients to regain their own renal function; however, severe shortage of organ donors and potential organ rejection limited its use. Consequently, exploring novel and better therapeutic options to alleviate, cure, or prevent renal diseases and to improve patients’ survival and quality of life is mandatory.

Stem cells (SCs) are described by their self-renewal abilities and the capability to develop into various functional cells under certain conditions. Based on the advantages of plasticity, infinite amplification, and ease of genetic manipulation, stem cell therapy opens new avenues for almost all human diseases. The application of SC therapy in treating a variety of diseases such as immunological, vascular, cardiac, and renal diseases has been extensively explored [6, 7]. The use of SCs is a promising therapeutic strategy for kidney diseases as well. Increasing results obtained in models of acute kidney injury (AKI) and chronic kidney disease (CKD) document that SCs have therapeutic potential in repair of renal injury, preserving renal function and structure thus prolonging animal survival. The effects were initially attributed to SCs implanting damaged tissue, differentiating, and replacing damaged cells [8]. The subsequent evidence suggests that SCs also act through the secretion of bioactive paracrine factors and/or release of microvesicles with immunomodulatory and proregenerative properties [9, 10]. Additionally, stem cells could be directed to differentiate into kidney lineage cells and generate into kidney organoids, which are expected to be used for disease modeling and drug discovery, and may eventually be applicable for transplantation [11]. In view of the great potential application of SCs in kidney diseases, the need to better understand on how to develop cell-based experimental treatments, and how to implement them in clinical trials, becomes more pressing. In this review, an overview of characteristics of different types of stem cells, the current available evidence from preclinical/clinical studies on cell-based therapy for kidney diseases, and discussion of prospects and challenges of its application in the treatment of kidney diseases were provided.

## Potential sources of stem cells for cell-based therapy in kidney diseases

**Fig. 1:**
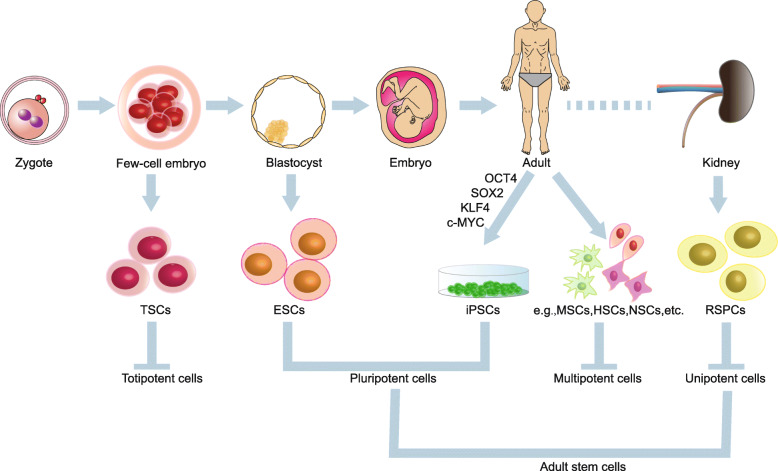
Classification of stem cells based on differentiation potential. TSCs, totipotent stem cells; ESCs, embryonic stem cells; iPSCs, induced pluripotent cells; MSCs, mesenchymal stem cells; HSCs, hematopoietic stem cells; NSCs, neural stem cells; RSPCs, renal stem/progenitor cells

Before we go into further details regarding the current SC-based approaches, a brief summary of the background information regarding potential sources of stem cells has to be put forward. SCs are self-renewable cells that are capable of differentiating into specialized cell types under appropriate conditions, and could be divided into four categories according to their differentiation potential (Fig. 1).

With the greatest differentiation potential, totipotent stem cells (TSCs) possess the omnipotentiality to differentiate into any type of cells, including the extraembryonic lineages and the germline [12, 13]. The zygote and the blastomeres of the early embryo are considered to be totipotent [14, 15]. Providing tools and models for exploring the underlying molecular mechanisms of cell potency, somatic cell nuclear transfer (SCNT) is currently the only way to generate a totipotent cell in vitro, which involved the physical transplantation of a somatic nucleus into an enucleated oocyte. Due to its association with low success rate and requirement of oocytes, there are both scientific and ethical considerations which hold back the broader practical use of this technology in stem cell therapy [16, 17]. But understanding TSCs helps us to understand the molecular mechanisms behind maintenance and manipulation of cell fate and is essential to understand how pluripotent cells form in vivo.

Pluripotency refers to a stem cell with potential to differentiate into any of the three germ lineages: endoderm (interior stomach lining, gastrointestinal tract, the lungs), mesoderm (muscle, bone, blood, urogenital), or ectoderm (epidermal tissues and nervous system) [18]. Pluripotent stem cells (PSCs) are capable of giving rise to all cells of any tissue types except for extraembryonic cells and mainly include embryonic stem cells (ESCs) and induced pluripotent stem cells (iPSCs). ESCs are harvested from the inner cell mass of blastocysts and are considered as the initial candidates of cell therapy [18, 19]. However, the clinical use of ESCs generated from surplus embryos has some problems, including bioethical debate, immunological rejections, and the risk of teratoma. One way to circumvent these issues is by generating PSCs directly from the patients’ own cells. Induced pluripotent stem cells are reprogrammed somatic cells that regain pluripotency by inducing a “forced” overexpression of certain transcription factors. iPSCs share many similarities with ESCs in terms of morphology, proliferation, surface markers, gene expression, epigenetic status, etc., and a less risk of immune rejection than ESCs. They are considered to be a potential source for renal generation and promising cell source for patients requiring specific cell therapy.

Adult SCs that exist in the postnatal organisms are either multipotent or unipotent and are restricted to a limited range of cell types if iPSCs were not taken into consideration. Multipotent SCs, including hematopoietic stem cells (HSCs) [20], mesenchymal stem cells (MSCs) [21], neural stem cells (NSCs) [22], and intestinal stem cells (ISCs) [23], can differentiate into several cell types of confined cell lineages. The MSCs, which are also known as the mesenchymal stromal cells (MSCs), can differentiate into cells of mesenchymal cell lineages such as osteocytes, adipocytes, and chondrocytes, and potentially other cell types [21]. In addition, MSCs were isolated virtually from any type of tissues, including the bone marrow stroma, adipose tissue, and umbilical cord blood. It has been hypothesized that MSCs contribute to tissue repair by differentiating into organ-specific cells and replacement of damaged tissues. Recently, a body of studies has confirmed that MSCs act through paracrine/endocrine secretion of bioactive factors and exosomes. MSCs secrete a variety of molecules including cytokines, growth factors, anti-oxidants and pro-angiogenic factors, and those that stimulate cell proliferation and angiogenesis, decrease the response to stress and apoptosis of damaged cells, and regulate the local and systemic inflammatory and immune response, thus contributing to tissue repair [24, 25]. The application of MSCs in treating kidney diseases is widely studied and is superior to the application of other SCs.

Progenitor cells exhibit differentiation potential between SCs and mature cells and were found in many organs, including the bone marrow [26], gastrointestinal mucosa [27], liver, brain, prostate, and skin. These cells play a part in maintaining the wounding process to replace the damaged or dead cells, participating in the normal cell turnover of organs. Whether or not adult kidney SCs exist has been an unsettled controversy to date, but current research has confirmed that the adult mammalian kidney contains multiple cells with self-renewability and differentiation potential, which are known as the renal stem/progenitor cells (RSPCs) [28–32]. With inherent renal differentiation potential and the potential for autologous treatments, RSPCs were considered to be ideal sources of cell therapy for treating kidney diseases.

## Embryonic stem cells

ESCs that are characterized by features of self-renewability and multilineage differentiation hold great promise in cell therapy and kidney regeneration. Numerous studies have shown that mouse embryonic stem cells (mESCs) can integrate into kidney compartments, suggesting a possible efficacy for kidney repair. In addition, the exposure of ESCs to factors required for renal specification, such as retinoic acid, activin A, and bone morphogenic proteins (BMPs), induces differentiation of these cells into renal lineage cells in vitro [33–35].

Geng et al. have reported that the ESC-loaded gelatin microcryogels on 5/6 nephrectomized rats slowed down the progression of chronic kidney disease and reduced glomerular injury [36]. Similarly, mESC implantation in mice with renal failure induction (RFI) employing cisplatin significantly decreased the mortality, avoiding a greater histological deterioration related to the disease. Glomerulosclerosis or collapsed glomeruli were not observed in RFI mice treated with mESCs; even some regeneration data were found characterized by large nuclei with prominent [37]. Mata-Miranda et al. suggested the beneficial effects of mESCs on AKI were related to the reduction of lipid peroxidation [38].

Patients require renal replacement therapy when their kidney disease progresses to ESRD. Although dialysis offers a temporary solution for patients, it has several limitations. Dialysis does not address the loss of hemostatic and endocrine function of the kidney and its associated complications [5]. Kidney transplantation remains to be the best curative treatment strategy to restore total kidney function, but the demand for organs suitable for transplantation has reached a level that far outstrips the static supply [39]. Due to the precise nature of renal structure, which comprises several kinds of cells and with complex anatomy, the kidney has become the most difficult organs of reconstruction. Many protocols have induced the differentiation of ESCs to generate complex structures resembling kidneys, termed organoids, which contain multiple renal cell types and are capable of self-organization.

Morizane et al. have successfully induced differentiation of ESCs into nephron progenitor cells (NPCs), which were then transformed into self-organized nephron-like structures containing glomeruli, proximal tubules, loop of Henle, and distal tubules in a contiguous, ordered arrangement that are analogous to that of nephrons [40]. Takasato and colleagues have generated kidney organoids that contain not only nephrons and collecting duct but also renal interstitium and an endothelial network. The proximal tubules of these show functional maturity to some extent [41]. The ESC-derived kidney progenitors implanted into immunocompromised mice generated perfused glomeruli containing human capillaries, podocytes with regions of mature basement membrane, and mesangial cells. The vascularized glomeruli showed the ability to produce ultrafiltrate that is processed by adjacent tubules [42]. The latest research is consistent with previous ones. By inducing the directional differentiation of ESCs into ureteric bud progenitor cells and co-culturing with dissociated primary metanephric mesenchyme (pMM), the organoids composed of nephron structures and collecting ducts could be produced, which displayed the presence of endothelial cells forming a vascular network [43].

Although scientists thought they have found a strategy to isolate hESCs from single blastomeres without destroying the embryos, legal and ethical controversies still limit the research and applications of ESCs [44]. Another concern about this technique is that cells and tissues derived from ESCs are at high risk of degenerating into neoplasms, especially teratomas. Yamamoto et al. demonstrated that ESCs can develop teratoma 14 days and 28 days after transplantation into mouse [45]. What is more, ESC-derived differentiated cells are allogenic in nature and inevitably suffer from all the issues related to allografts and immunocompatibility, such as acute and chronic rejection, and graft versus host disease.

## Induced pluripotent stem cells

Induced pluripotent stem cells sharing many of the regenerative properties of ESCs are considered effective alternative cell sources for ESCs. Indeed, iPSCs could retain both genetic background and peculiar epigenetic memory of the cells of origin, providing indisputable advantages in cell therapy, kidney regeneration, and other biomedical applications.

According to the earliest report on iPSCs put forward in 2006, scientists obtained PSCs by introducing four transcription factors (OCT4, SOX2, KLF4, and c-MYC) into mouse adult fibroblasts, and this technique has earned Shinya Yamanaka and John Gurdon the Nobel Prize in Physiology or Medicine 2012 [34]. To date, the human iPSCs have been generated from multiple sources, including skin fibroblasts, keratinocytes [46, 47], extraembryonic tissues [48], cord blood [49], peripheral blood cells [50], hepatocytes, stomach cells [51], dental pulp cells [52], and even fully differentiated lymphocytes (such as T and B cells) [53]. Interestingly, the terminally differentiated kidney cells are reprogrammed to pluripotency. Song et al. have reprogrammed normal human mesangial cells to pluripotency by retroviral transduction using defined factors (Oct4, Sox2, Klf4, and c-Myc). The kidney-derived iPSCs resemble that of human embryonic stem cell-like colonies in morphology and gene expression. They were considered as alkaline phosphatase-positive; express OCT3/4, TRA-1 to 60, and TRA-1 to 81 proteins; and form embryoid bodies and express markers of all three germ layers [54]. iPSCs were generated from human renal proximal tubular cells as well. Considering their potential risks as oncogenes Klf4 and c-Myc, Montserrat et al. have generated iPSCs with only two transcription factors, Oct4 and Sox2, by using a single tricistronic vector and without any need of other additional chemical compounds [55]. Moreover, generation of iPSCs from exfoliated renal tubular cells that are present in urine is regarded as a simple, non-invasive method for obtaining iPSCs [56–58]. Indeed, iPSCs could retain both genetic background and peculiar epigenetic memory of the cells of origin, providing indisputable advantages in cell therapy, kidney regeneration, and other biomedical applications.

Induced pluripotent stem cells are considered as an effective means to alleviate renal tissue damaged in AKI and CKD, and paracrine pathways may be the major mechanism. The administration of iPSC-derived conditioned medium attenuated AKI by downregulating the oxidative stress response in ischemia-reperfusion rats [59]. The recent result from Collino et al. suggested that iPSC-derived extracellular vesicles contributed to the reduction of macrophage infiltration and protected functional mitochondria and regulated several genes associated with oxidative stress, promoting the renoprotection of AKI rats [60]. In the study conducted by Lee et al., the cell transplantation of iPSCs showed improvement of renal function after AKI and dramatically reduced the mortality associated with AKI, exhibiting anti-oxidative stress, anti-inflammatory, and anti-apoptotic effects. It is worth noting that high doses of iPSC accumulation might lead to renal dysfunction. In the same research, Lee et al. have found that injection with 5 × 10^7^ iPSCs affected the smaller arteries of the kidneys with cellular emboli, leading to the reduction of blood reperfusion after ischemia. When carrying out cell therapy for treatment of AKI using iPSCs, the cell dose for transplantation and the monitoring of renal blood perfusion should be taken care of [61]. For the efficacy of iPSC therapy in chronic kidney disease, Caldas et al. have compared the therapeutic effects of iPSCs and bone marrow-derived mesenchymal stromal cells (BM-MSCs) on the progression of CKD in untreated 5/6 nephrectomized rats. It was shown that both treatments improved function and structure of the kidney. Injection of iPSCs decreased the macrophage infiltration, and TGF-β was reduced in the kidneys in the BMSC group, suggesting an anti-inflammatory response that led to decreased fibrosis. In particularly, proteinuria was reduced only in the iPSC group. However, 5/8 (62.5%) rats treated with iPSCs presented tumors confirmed by histology, the cells are positively stained by PCNA, and Wilms’ tumor protein antibody has the characteristics of Wilms’ tumor. These results indicated the effect of iPSCs on retarding progression of CKD and potential risk of Wilms’ tumor development [62].

The pluripotency of PSCs raises concerns on high risk of maldifferentiation of the cells and even tumor formation, when these cells are administered without pre-differentiation. An attractive alternative consists in the derivation of renal progenitors from iPSCs, which could be achieved by the controlled activation of the correct network of nephric transcription factors. The injected iPSC-derived renal progenitor cells robustly engrafted into damaged tubuli and restored renal function and structure in cisplatin mice with AKI [63]. Similarly, iPSC-derived MSC therapy effectively protected the rat kidney from acute ischemia-reperfusion injury [64]. And iPSC-derived MSCs showed comparable effects in the repair of AKI as compared to adult MSCs [65]. In CKD rat models, the administration of iPSC-derived MSCs selectively mobilized into kidney parenchyma and preserved residual renal function via suppression of the inflammatory reaction, inhibiting apoptosis and regulating cell proliferative/death signaling. All the studies above demonstrate that iPSCs are a valuable source of engraftable cells with regenerative activity for kidney disease and create the basis for future applications in stem cell-based therapy [66].

Induced pluripotent stem cells are regarded as the best source for producing new kidney tissues for transplantation, which are potentially derived from patients and used as a renal replacement therapy without immunosuppression. Although it is difficult to regenerate the entire kidney in vitro, recent advances in the field of SCs have enabled in vitro generation of organoids [67–73].

The kidney organoid formation involves stimulation of a stepwise differentiation process, in which movement occurs from monolayered iPSCs to primitive streak then intermediate mesoderm to kidney lineage cells [40, 69–71, 74]. Several protocols for differentiation of PSCs to kidney cell fates have been established to date. In the early days, several studies have reported successful differentiation of human pluripotent stem cells (hPSCs) into either ureteric epithelium [35, 73, 75] or metanephric mesenchyme [35, 72, 76] in vitro. Takasato et al. have put forward a detailed protocol of inducing human induced pluripotent stem cells (hiPSCs) to generate complex multicellular kidney organoid within which segmented nephrons are connected to the collecting ducts and are surrounded by renal interstitial cells and an endothelial network. The kidney organoid reported is transcriptionally similar to that of fetal human kidneys and showed functional maturity, in which the proximal tubules within the organoids displayed megalin-mediated and cubilin-mediated endocytosis, and responded to a nephrotoxicant to undergo apoptosis [70].

Bioengineering represents one possible approach for generating the complex branching structures of the kidney. Scaffolds are temporary physical support obtained from a variety of biomaterials and help to accommodate cells and support their three-dimensional (3D) growth during tissue developmental stage [77]. Scaffolds have become a hope for clinical translation due to their features of biocompatibility, biochemical and biological cues for cell adhesion, proliferation, migration, differentiation, and continued function [78, 79]. Renal scaffolds were successfully produced from porcine, rat, and human kidneys [80–84]. Considering as a potent cell source for scaffold recellularization, embryonic stem cells and adipose tissue-derived stem cells have been seeded into renal scaffolds, achieving cell adherence, proliferation, differentiation, endothelialization, and vascularization [84–87]. Du et al. have first attempted to repopulate a kidney scaffold using hiPSC-derived endothelial cells (hiPSC-ECs) [88]. Ciampi et al. have repopulated acellular rat kidney scaffolds by using hiPSC-ECs that properly integrate into kidney structures, forming vessel-like structures [89]. It has been reported that human whole kidney scaffold re-endothelialization can be done using hiPSC-ECs and fully perfused with human whole blood [84]. Microfluidic organ-on-a-chip technology has been used to construct an in vitro model of human kidney glomerulus. Musah et al. have reconstituted kidney glomerular capillary function in vitro through microfluidic organ-on-a-chip device. The hiPSC-derived podocytes were cultured on the top of the laminin-coated membrane and primary human glomerular endothelial cells on the opposite side of the same membrane to recapitulate the podocyte-GBM-endothelial interface. The microfluidic glomerulus chip recapitulates some of the normal molecular filtration properties of the functional human kidney glomerular capillary wall and then replicates pharmacologically induced podocyte injury and albuminuria as seen in patients [90, 91].

Although not yet clinically available for transplantation, hiPSC-derived kidney organoids provide dominant models for studying the pathophysiology of kidney diseases, through which we can better understand gene mutations and disease phenotypes of the disease, mimicking disease progression. Preserving the naturally occurring genetic mutations as well as the genetic background of their parental somatic cells, hiPSCs can be applicable to disease/patient-specific modeling. Genetically characterized hiPSCs have been efficiently derived from patients with autosomal dominant polycystic kidney disease (ADPKD) and autosomal recessive PKD (ARPKD), Alport syndrome, systemic lupus erythematosus, and Wilms’ tumor and patients undergoing hemodialysis [58, 92–97]. In addition, mouse models commonly used in previous studies could not fully recapitulate human genotypes and phenotypes [98, 99]. For example, KAL-1 encodes a basement membrane protein called anosmin-1 that is present on the surface of ureteric bud (UB). The mouse lacking KAL-1 and humans with KAL-1 mutations have renal agenesis, absence of kidneys and ureters [100]. Another case is that the autosomal dominant polycystic kidney disease in humans often inherits heterozygous loss-of-function mutations in either PKD1 (polycystic kidney disease-1) or PKD2 (polycystic kidney disease-2), while the mice displays only very mild cystic disease in the same situation [101, 102]. Species-specific models developed by hiPSCs that carry naturally occurring human mutations are regarded as important complement to mouse models, showing a great promise in studying kidney development and disease process.

Recently, gene editing technologies such as CRISPR/Cas9 make it possible to treat inherited kidney diseases by correcting the pathogenic mutation in an iPSC line. For example, a family with adult-onset autosomal dominant focal segmental glomerulosclerosis (FSGS) was recently found to carry a new germline missense heterozygous mutation (p.G189R) in the octapeptide domain of the transcription factor PAX2 [103]. Trionfini et al. efficiently corrected this point mutation in patient-derived iPSCs by means of CRISPR-Cas9-based homology-directed repair. Forbes et al. corrected the mutations in IFT140 of iPSCs derived from a patient with nephronophthisis-related ciliopathy and induced the isogenic gene-corrected iPSCs differentiated to kidney organoids. Proband organoid tubules demonstrated shortened, club-shaped primary cilia, whereas gene correction rescued this phenotype [104]. Likewise, genetic correction of the single amino acid mutation of iPSCs generated from a congenital nephrotic syndrome patient due to NPHS1 mutations has restored nephrin localization and phosphorylation and slit diaphragm formation [74].

## Mesenchymal stem/stromal cells

**Fig. 2:**
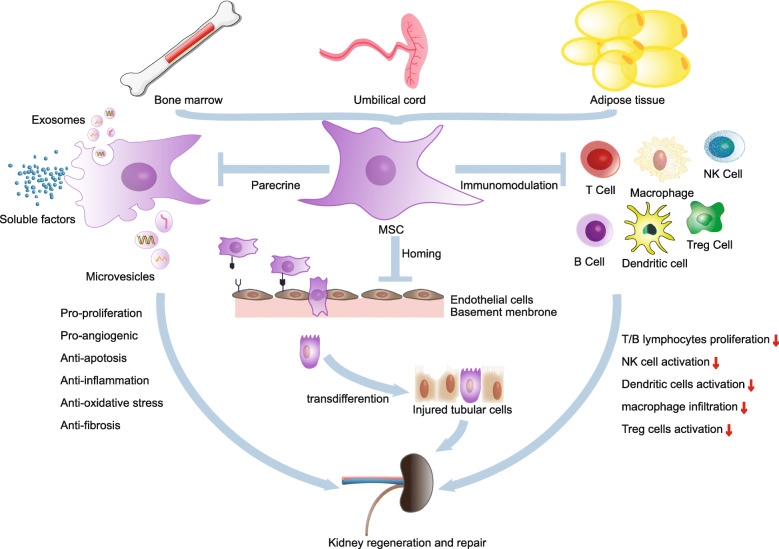
The diverse mechanism of MSCs in the treatment of kidney diseases

Besides the therapies based on HSCs, MSC-based therapies are currently the most advanced SC-based therapies in terms of clinical testing. Firstly, MSCs can be readily expanded in culture, generating a large number of therapeutic doses. Secondly, MSCs do not express blood group, DR antigens, and costimulatory CD40, CD80, and CD86 proteins to render them hypoimmunogenic, thus facilitating their safe use in allogeneic off-the-shelf protocols [105]. Accumulating pieces of evidence have indicated tremendous treatment potential of MSCs in kidney diseases, including AKI, CKD, diabetic nephropathy (DN), lupus nephritis (LN), polycystic kidney disease (PKD), and renovascular disease (RVD). Although a uniform mechanism governing the MSC-based therapy has not yet been discovered, available data have revealed several working models that promoted their use (Fig. 2).

The priority of MSCs to traffic to the sites of ischemia, hypoxia, and inflammatory response following injury plays a crucial role in the success of these as cellular therapy for organ injuries. MSC homing is defined as the arrest of MSCs within the vasculature of a tissue followed by transmigration across the endothelium [106–108]. The homing of MSCs to the injured tissues has been driven by a combination of chemokine release from the injured tissue and chemokine receptors that are expressed by MSCs [109]. The stromal derived factor-1 (SDF-1)/chemokine (C-X-C motif) receptor 4 (CXCR4) axis plays a crucial role in the migration of MSCs. Overexpression of CXCR4 gene enhances BM-MSC migration to the kidney area and functional repair after AKI [110]. CD44-hyaluronic acid (CD44-HA) interactions are involved in the process of homing of MSCs to the damaged renal tissue and promote renal function repair following acute tubular injury and chronic renal failure [111]. Due to their vitality in enhancing the kidney-directional migration of transplanted MSCs for increasing the efficiency of tissue repair, some novel preconditioning strategies were explored, including incubation with cytokines or chemical compounds [112–114], co-injection [115, 116], hypoxia stimulation [117], and genetic modifications [118, 119]. Also, pulse-focused ultrasound (pFUS) has elicited local molecular responses through mechanotransduction, thus enhancing the renal homing of circulating MSCs [120–122].

Treatment with intravenous injection of MSCs has efficiently induced improvement of the repaired kidney both morphologically and functionally. Therapeutic properties of MSCs were originally derived from their engraftment in the injured kidney and subsequent transdifferentiation of MSCs into renal-specific cells to repopulate the kidney. The possible role of MSCs in response to kidney injury was first observed when the female kidney transplants were transferred into male recipients, in which the Y chromosome staining of tubular epithelial cells was found [123, 124]. Several studies have shown that intravenous injection of MSCs homed to the repaired kidney site and localized in the context of tubular epithelial lining and expressed the epithelial markers, indicating engraftment of MSCs [8, 109, 125, 126]. Although MSCs home to the injured sites of kidney via blood circulation after transplantation, the renal MSC engraftment was scarce and transient. Most of injected MSCs remain in other blood-rich organs, such as the lung, liver, and spleen. The numbers of MSC-derived epithelial cells appear to be so low to explain tissue recovery or wound healing (≤ 0.1%), and the hypothesis could not explain the rapid protective effect of injected MSCs which occurred within 24–48 h as well. All these facts suggest that the effects of MSCs may be mediated by another mechanism. The research conducted by Tögel et al. has made a breakthrough in this field. The study reported that differentiation of rat BM-MSCs into a tubular or endothelial cell phenotype was not observed in an ischemic/reperfusion experimental model of AKI. But the expression of proinflammatory cytokines IL-1β, TNF-α, IFN-γ, and inducible nitric oxide synthase showed significant reduction, and that of anti-inflammatory factors IL-10 and bFGF, TGF-α, and Bcl-2 was highly upregulated in the treated kidneys [127]. These studies showed consistent results, in which MSC-conditioned media improved the survival of human proximal tubular cells when exposed to cisplatin in vitro [10, 128, 129]. Now, there is a growing consensus that kidney-protective effects of MSCs are primarily exerted by their paracrine function rather than by direct engraftment.

MSCs secrete a broad repertoire of cytokines, chemokines, and growth factors, including IL-6 and IL-11, vascular endothelial growth factor (VEGF), granulocyte-colony stimulating factor (G-CSF), macrophage-colony stimulating factor (M-CSF), basic fibroblast growth factor (bFGF), monocyte-chemoattractant protein-1 (MCP-1), insulin-like growth factor (IGF), leukemia inhibitory factor (LIF), and hepatocyte growth HGF [10, 130–133]. These contribute in renal protection by promoting proliferation of epithelial cells and angiogenesis, anti-apoptosis, anti-inflammatory, anti-fibrosis, and other signaling pathways [10, 134–136]. Moreover, accumulating evidence also underlined the paracrine actions mediated by extracellular vesicles (EVs), small anuclear membrane-bound particles released from MSCs as a paracrine vehicle to deliver messenger RNA (mRNA), microRNAs, proteins, or bioactive lipids that may reprogram the injured cells or induce secretion of cytoprotective factors [137, 138]. The EVs mainly include exosomes (30–100 nm) that are formed from multivesicular bodies through inward budding of endosome membrane [139] and microvesicles (100–1000 nm) that are released from the cell by outward budding of plasma membrane [140]. Indeed, it has been shown that MSC-derived EVs (MSC-EVs) accelerate renal repair in acute kidney injury (AKI) models. Bruno et al. have demonstrated the protective effects of MSC-EVs in tubular epithelial cells of severe combined immunodeficient (SCID) mice in glycerol-induced AKI. RNase treatment abrogated the protective effects, suggesting an RNA-dependent mechanism and the occurrence of horizontal transfer of human mRNAs in MSC-EVs to target cells [141]. Similar results were obtained by Gatti et al. [142] and Zou et al. [143]. Injections of MSC-EVs reduced the progression of renal injury in experimental models of CKD that was induced by 5/6 nephrectomy [144] and unilateral ureteral obstruction [145, 146]. Eirin and his colleagues have tested the therapeutic effects of EVs in a porcine model with coexistence of RVD and metabolic syndrome. Intrarenal delivery of MSC-EVs attenuated renal inflammation; improved medullary oxygenation and fibrosis; decreased renal vein levels of several pro-inflammatory cytokines, including TNF-α, IL-6, and IL-1-β; and upregulated anti-inflammatory cytokine IL-10 [135]. Moreover, MSC-derived EVs inhibited and reverted fibrosis progression in a diabetic nephropathy mouse model by downregulating fibrosis-related genes [147].

Rather than being a constant mixture of molecular factors, MSCs’ secretome is known to be dependent on the diverse stimuli present in the microenvironment that MSCs encounter. The crosstalk and interplay of MSCs and local environment reversely control and regulate the paracrine activity of MSCs. MSC growth conditions such as oxygen tension, growth factor composition, and mechanical properties may serve to directly influence paracrine activity [148, 149]. Daniëlle et al. cultured human bone marrow and kidney perivascular stromal cells in the TopoWell plate, a custom-fabricated multiwell plate containing 76 unique bioactive surface topographies. Then, they compared the 76 different surface topographies in growth factor and cytokine expression profile; major differences can be observed between secretion levels between topographies of several growth factor and cytokine levels, such as HGF, SDF1α, and trombospondin-I, while others showed a more stable secretion such as VEGF [150]. As the paracrine of MSCs is affected by microenvironment structure, the therapeutic potential of their secretome can be manipulated in an engineered setting. For example, BM-MSCs preconditioned with melatonin express a higher level of catalase and SOD-1 expression and overexpression of basic fibroblast growth factor (bFGF) and HGF. And more surviving grafted MSCs, overstimulation of angiogenesis and proliferation in renal cells, and more rapid renal function recovery were observed in I/R-AKI rats which received the BM-MSCs preconditioned with melatonin [151].

MSCs comprise immunomodulatory and tolerogenic properties. Extensive in vitro and in vivo studies have demonstrated that MSCs are capable of suppressing T cell proliferation, influencing dendritic cell maturation and function, suppressing B cell proliferation and terminal differentiation, and modulating other immune cells such as natural killer (NK) cells and macrophages. Besides, MSCs also induce regulatory T cells (Tregs) and maintain the capability of Tregs to suppress self-reactive T-effector responses [152]. The immunosuppressive capacity of MSCs allows them to act as a potential therapeutic target in renal transplantation as they may inhibit allograft rejection and induce transplant tolerance [153, 154]. On the other hand, immunological properties of MSCs suggest a potential treatment for autoimmune diseases, such as lupus nephritis characterized by autoantibody production and immune complex formation/deposition in glomeruli. A significant reduction in the levels of anti-dsDNA and anti-nuclear autoantibodies, immunoglobulin IgG, IgM, and serum albumin was observed in SLE mice after BM-MSC therapy. And BM-MSC transplantation effectively prevented damage to glomerular morphology/structure and reduced renal complex deposition of both complement component 3 (C3) and IgG [155]. The study conducted by Jang et al. showed that the infusion of BM-MSCs suppressed glomerulonephritis, decreased autoantibody production, reduced proteinuria, and improved overall survival in SLE mice via inhibition of follicular helper cell (Tfh) development and activation of humoral immunity [156]. Dental tissue-derived MSCs are also effective in reducing kidney glomerular lesion and perivascular inflammation infiltration [157]. However, some studies failed to show a positive effect of the treatment with MSCs, and for some aspects, even deleterious results seem to be observed [158].

Despite promising preclinical results obtained in animal models, which indicated effectiveness of MSCs in reducing acute and chronic kidney injuries, clinical trials still remained in the early phases and largely aimed to investigate the safety and efficacy of MSC infusion. To date, more than 40 clinical trials were conducted worldwide, completed or ongoing, involving the use of MSCs in the treatment of kidney diseases as reported by the US National Institute of Health database (ClinicalTrials.gov).

According to the clinical study conducted by Gooch et al., infusion of allogenic MSCs into patients with high risk of AKI after undergoing on-pump cardiac surgery demonstrated no adverse events (AEs) or severe adverse events (SAEs). Moreover, MSC infusion prevented postoperative renal failure (0% versus 20% AKI incidence when compared to case controls) and the length of hospital stay and readmission rates were reduced by 40% [159, 160]. A recent phase 2, randomized, double-blind, placebo-controlled trial conducted by Swaminathan et al. drew inconsistent conclusions to those stated above. No significant difference was observed in the time to recovery of kidney function after treatment of early postoperative AKI with allogeneic MSCs when compared to those treated with placebo [161]. The absence of a significant recovery signal after AKI in patients treated with MSCs might be attributable to several factors. Longer durations of median cardiopulmonary bypass (CPB) time in the MSC group might have masked a beneficial effect. Many patients in this study had impaired kidney function before surgery, and in the setting of already compromised kidney function, beneficial effects of MSCs could be attenuated. What is more, the complexity of postcardiac surgery clinical setting and postoperative course might hinder the ability to observe a modest clinical effect with MSCs or other agents.

Although clinical translation of MSC-based therapeutics for human CKD is still in an early phase, it has demonstrated the potential to modify the progression of CKD. A single-center, randomized, placebo-controlled, phase II/III clinical pilot study investigated the safety and therapeutic efficacy of MSC-derived EVs in CKD patient stage III and IV after following up for a period of 12 months. Although kidney biopsy specimens that are obtained 3 months after the therapy showed no significant histologic changes, EV treatment exhibited a significant improvement in renal function (improved eGFR and decreased serum creatinine, BUN, and albuminuria) [162]. A multicenter, randomized study demonstrated the safety of allogeneic mesenchymal precursor cell (MPC) infusion in diabetic nephropathy. DN patients with eGFR of 20–50 ml/min/1.73 m2 were treated with a single intravenous infusion of two doses of allogenic MPC or placebo. Relative to placebo, a single IV rexlemestrocel-L infusion showed trends of stabilizing or improving eGFR and measured glomerular filtration rate (mGFR) at the prespecified primary endpoint 12 weeks post-infusion, particularly for those with higher baseline eGFR (> 30 ml/min/1.73/m2) and higher serum IL-6 concentration (> 3.5 pg/dl) [163]. In another pilot study, the treatment of autologous adipose tissue-derived MSCs (AT-MSCs) significantly reduced urinary protein excretion of CKD patients, from a median of 0.75 g/day (range, 0.15–9.57) at baseline to 0.54 g/day (range, 0.01v2.66) at month 12. But no improvement in glomerular filtration rate has been observed [164]. Similarly, another 18-month study showed no significant improvements in renal function of CKD patients who received MSC infusion [165]. Saad et al. have reported that intra-arterial infusion of autologous MSCs in patients with RVD showed increased cortical perfusion and renal blood flow (RBF) and reduced renal tissue hypoxia within post-stenotic kidney [166]. The safety and tolerability of a single BM-MSC intravenous infusion in ADPKD patients were observed [167].

The safety and tolerability of MSCs in patients with LN have also been described. Numerous clinical studies have shown that allogeneic MSC transplantation in patients with SLE resulted in the amelioration of disease activity, improvement in serological markers, and stabilization of renal function [168–171]. A single-center study involving 81 Chinese patients with active and refractory LN reported that 60.5% (49/81) patients have achieved renal remission during 12-month visit by human umbilical cord-derived mesenchymal stem cell (hUC-MSC) transplantation, and 11 of 49 (22.4%) remission patients experienced renal flare at the end of 12 months [172]. Similar findings have been obtained in the research conducted on 3 patients with class IV LN who were treated with allogeneic MSCs. Two patients had early, durable, and substantial complete remissions, while the third patient achieved a partial remission, which permitted reduction of medication doses by 50–90%. Proteinuria levels were improved dramatically during the first month after treatment, and the ameliorations were sustained throughout the 9-month follow-up period [173]. In contrast, a recently published multicenter, randomized, double-blind, placebo-controlled clinical trial in 18 patients with World Health Organization class III or IV LN failed to show any beneficial effect of hUC-MSCs [174].

Given the immune-regulatory properties, MSCs have been administered to kidney transplant recipients, with the aim of controlling the host immune response towards the graft and minimizing immunosuppression, possibly leading to transplant tolerance. A previous study conducted by Perico et al. have reported that 2 recipients of kidney transplant from a living-related donor treated with autologous bone marrow-derived MSC autologous (BM-MSCs) have had transient renal insufficiency (engraftment syndrome) 7–10 days after single infusion [175]. Kidney biopsy revealed infiltration of immune cells with C3 deposits. It is hypothesized that kidney transplantation triggered graft inflammation, causing recruitment of MSCs to the graft and favoring differentiation of MSCs towards a proinflammatory phenotype [176]. Perico et al. have amended the clinical protocol to plan MSC infusion the day before transplantation, and two subsequent patients who received MSCs before transplantation did not experience any engraftment syndrome. Infusion of MSCs has promoted the development of a pro-tolerogenic milieu, which is characterized by an increase in the ratio between Tregs and memory CD8+ T cells in the peripheral blood as well as a profound reduction in ex vivo anti-donor CD8+ T cell cytolytic function. All MSC-treated patients had stable graft function during a 5–7-year follow-up, without increasing the susceptibility to infections or malignancy [177, 178]. Erpicum et al. reported that MSC-treated recipients showed increased frequencies of regulatory T cells on day 30, with no significant change in the B cell frequencies when compared to that of concurrent controls [179]. Clinical researches conducted by Mudrabettu et al. or Reinders et al. were in line with the results of the above research [180, 181]. Notably, administration of MSCs has the potential to reduce the usage of immunosuppressants. Mounting evidence indicates that donor-derived MSCs when combined with low-dose tacrolimus have similar efficacy to that of standard immunosuppression following living-related renal transplantation, allowing 50% reduction of maintenance immunosuppression by calcineurin inhibitors (CNI) [182]. A large randomized clinical trial showed that among patients undergoing renal transplantation, the use of autologous MSCs when compared with anti-IL-2 receptor antibody induction therapy resulted in lowering the incidence of acute rejection, decreasing the risk of opportunistic infection, and better estimated renal function at 1 year follow-up [183] (Table 1).

**Table 1:** Registered clinical trials testing the efficacy of MSCs in kidney

Recipients	Source	Administration route	Primary endpoint	Secondary endpoint	Dose	Follow-up period	Trial registration	Ref.
**AKI**								
Patients with high risk of developing AKI following heart surgery	Allogeneic BM-MSCs	Intra-aortic	Safety, as documented by comparing the incidence of adverse events, serious adverse events, and complications	Efficacy of MSC administration for prevention and treatment of postoperative AKI	NA	6 months	NCT00733876	[159, 160]
Patients undergoing cardiac surgery with evidence of early AKI	Allogeneic MSCs	Intra-aortic	The time to recover kidney function was defined as return of postintervention creatinine levels to baseline	Included all-cause mortality or provision of dialysis at 30 and 90 days post-study drug administration	2 × 10^6^cells/kg	90 days	NCT01602328	[161]
**CKD**								
CKD patients (eGFR 15–60 mg/ml)	Supernatants of hCB-MSCs	Intravenous and intra-arterial	The safety of the therapy through for a period of 1 year	The efficacy of treatment assessed by duplication of eGFR or 50% reduction of serum creatinine from the baseline level of each patient	100 μg/kg	12 months	NA	[162]
Type 2 DN patients	Allogeneic BM-MPCs	Intravenous	The safety and tolerability, including the number and percentage of subjects with adverse events and serious adverse events, clinically significant values and shifts from baseline in vital signs, and clinical laboratory tests	Efficacy change from baseline in eGFR and directly measured GFR by 99Tc-DTPA plasma clearance at 12 weeks post-infusion	150 × 10^6^ or 300 × 10^6^ cells/kg	60 weeks	NCT01843387	[163]
CKD patients (eGFR 20–40 ml/min/1.73 m^2^)	Autologous AT-MSCs	Intravenous	The eGFR and quantitative 24-h urinary protein excretion rate for a period of 12 months	Clinical or biochemical changes suggestive of treatment-associated adverse events or warnings	1 × 10^6^ cells/kg	12 months	NA	[164]
CKD patients (eGFR 25–60 ml/min/1.73 m^2^)	Autologous BM-MSCs	Intravenous	The safety was measured by number and severity of adverse events	Decrease in the rate of decrease in eGFR	1–2 × 10^6^ cells/kg	18 months	NCT02195323	[165]
RVD patients	Autologous AD-MSCs	Intra-arterial	1. Change in kidney function 2. Safety of mesenchymal stem cell infusion	Decrease in kidney inflammation	1.0 × 10^5^ or 2.5 × 10^5^cells/kg	3 months	NCT02266394	[166]
ADPKD patients	Autologous BM-MSCs	Intravenous	The numbers, type, and severity of AEs	Changes in eGFR from baseline to 12 months after cell infusion	2 × 10^6^ cells/kg	12 months	NCT02166489	[167]
Patients with active and refractory LN	Allogeneic BM-MSCs (*n* = 23) or hUC-MSCs (*n* = 58)	Intravenous	Remission of nephritis (CR and PR) as well as renal flares within 12 months	SLEDAI score for disease activity, BILAG score of renal system, and changes in renal function, serum albumin, and anti-dsDNA antibody levels pre- and post-MSCT	1 × 10^6^ cells/kg	12 months	NA	[173].
Patients with class III or IV LN	hUC-MSCs	Intravenous	Remission of nephritis was defined as stabilization or improvement in renal function, urinary red blood cells of less than 10 per hpf, and reduction of proteinuria to less than 3 g/day if baseline proteinuria was more than 3 g/day or at least a 50% reduction in proteinuria or to less than 1 g/day if baseline proteinuria was in the subnephrotic range	Improvement in SLEDAI and BILAG scores, complement concentration, anti-dsDNA (double-stranded DNA) antibody and ANA titers, death and commencement of permanent dialysis or renal transplantation	2 × 10^8^ cells/kg	12 months	NCT01539902	[174]
**Renal transplantation**								
Kidney transplant recipients	Autologous BM-MSCs	Intravenous	Assessing the percentage of inhibition of memory T cell response and/or naive T cell response, the induction of donor-reactive T cell anergy, and the appearance in the peripheral blood of regulatory T cells	Safety parameters related to MSC infusion, graft function, graft rejection	2 × 10^6^/kg	1 year	NCT00752479	[175, 177]
Kidney transplant recipients	Allogeneic BM-MSCs	Intravenous	Adverse effects of MSC infusion as well as infectious and malignant complications at 1 year	Effect of MSCs on graft outcomes and immunity as well as the occurrence of anti-MSC-DSAs	1.5 × 10^6^–3 × 10^6^ cells/kg	1 year	NCT01429038	[179]
Kidney transplant recipients	Autologous BM-MSCs	Intravenous	Changes of regulatory T cells and serum creatinine	Changes T cell proliferation, regulatory T cells, memory T cells, B cells, and cytokine profile	0.2–0.8 × 10^6^/kg	6 months	NCT02409940	[180]
Kidney transplant recipients	Autologous BM-MSCs	Intravenous	1. Rate of (serious) adverse events in the study population 2. Feasibility: determination of the number of expanded MSCs in relation to the amount of BM collected, number of passages required, and time to reach study target doses	1. Presence of late acute rejection (at 6-month biopsy when compared with 4-week biopsy) 2. Sirius red staining for renal cortical matrix accumulation. 3. Immunologic response before and after MSC infusion	1 × 10^6^ cells/kg	24 weeks	NCT00734396	[181]
Kidney transplant recipients	Autologous BM-MSCs	Intravenous	1-year incidence of acute rejection and renal function eGFR	Patient and graft survival and incidence of adverse events	1–2 × 10^6^ cells/kg	30 months	NCT00658073	[183]

*AD-MSCs* adipose-derived mesenchymal stem cells, *ADPKD* autosomal dominant polycystic kidney disease, *AEs* adverse events, *anti-MSC-DSAs* anti-mesenchymal stem cell donor-specific antibodies, *AT-MSCs* adipose tissue-derived MSCs, *BM-MSCs* bone marrow-derived mesenchymal stromal cells, *BM-MPCs* bone marrow-derived mesenchymal precursor cells, *CR* complete remission, *DN* diabetic nephropathy, *eGFR* estimated glomerular filtration rate, *hUC-MSCs* human umbilical cord-derived mesenchymal stem cells, *hCB-MSCs* human cord blood mesenchymal stem cells, *LN* lupus nephropathy, *MPCs* mesenchymal precursor cells, *NA* not applicable, *PR* partial remission, *RVD* renovascular disease, *SAEs* severe adverse events

## Renal stem/progenitor cells

It is widely accepted that embryonic renal SCs with nephrogenic potential would deplete soon after (mice) or several weeks before (humans) birth. Interestingly, at least 6000 cells derived from different nephron segments are lost every hour in the urine, suggesting that the progenitor cells are required for continuous replacement of loss of cells during physiological processes [184]. Additional evidence from several studies indicated that adult kidney upon acute or chronic injury has some ability to survive injury and undergo structural remodeling or repair, supporting that it has certain potential to retain regeneration. These have greatly encouraged researchers to search for resident renal stem/progenitor cells (RSPCs).

To our knowledge, there is no single bona fide marker specific for RSPCs at present. Various attempts have been made to identify and characterize RSPCs from differentiated cells in adult kidney, including label-retaining assays, flow cytometry using cell surface markers, and side population assays.

In general, SCs have a slow turnover rate and display minimal physiologic differentiation. When labeled nucleic acids were incorporated, BrdU or 3H-thymidine, SCs retain the label for longer periods of time, while other cells lose [185]. Label-retaining cells (LRCs) are believed to represent the SC compartment. Maeshima and colleagues have first demonstrated the existence of LRCs in renal tubuli of normal rat kidneys. Oliver et al. have identified LRCs in the mouse papilla. These cells form spheres in vitro as well as potentially differentiate towards other lineages [186]. LRCs formed tubule-like structure in 3D culture system and, when LRCs were injected into cultured metanephros, formed nephrons and collecting ducts [187]. The drawback of this technique is that the labels such as BrdU might be released from the dying cells and were taken up from the adjacent dividing cells [188].

By using SC marker expression analyses, the RSPCs were identified in the proximal tubules, in the medullary papilla region, and in the Bowman capsule of human kidneys, which are commonly characterized by the expression of CD133^+^ and embryonic renal marker PAX2 [30, 189, 190]. Further researches have suggested that CD133^+^ renal stem cells always co-express CD24 in adult human kidney tissue [191]. The CD24^+^CD133^+^ cells had self-renewability and pluripotent differentiation potential, forming a subset of parietal epithelial cells (PECs) in Bowman’s capsule by expressing SC-specific transcription factors Oct-4 and BmI-1 [28]. Numerous studies have demonstrated that CD24^+^CD133^+^ cells from adult human kidney represent a multipotent adult resident stem cell population [31, 191–193]. It also has been reported that CD24^+^CD133^+^cells could be isolated from human urine, which exhibit the same phenotypic and functional features of RSPCs obtained from human kidney tissue and show the potential to differentiate into tubular cells as well as podocytes [194]. Moreover, the RSPCs were further characterized molecularly by expressing the embryonic transcription factor OCT4 and renal developmental genes PAX2, SIX2, and SALL1, and low expression of fully differentiated kidney cell markers [195, 196].

Other selection strategies based on functional assays were utilized to identify and select RSPCs from human renal tissue. With the ability to effectively efflux the Hoechst dye, RSPCs can be detected via flow cytometry and are termed as the side population (SP) cells, which were found in murine kidney interstitial space in several groups [197–200]. Aldehyde dehydrogenase (ALDH) activity was used to isolate cells with progenitor characteristics from adult human renal cortical tissue. The ALDH_high_ cells expressed CD24 and CD133 and displayed typical SC properties such as sphere formation and anchorage-independent growth [29]. Although these cells were identified using a different strategy and were localized in different nephron segments, these all exhibited the capacity to self-renew and the ability to differentiate towards renal tubules or podocytes and different lineages.

Experiments in rat and mouse models of tubular or glomerular damage suggested a therapeutic effect of adult RSPCs of different types and sites of origin. The mechanism of RSPCs involved in kidney repair is associated to integrate into nephrons and is in parallel to a paracrine mechanism based on renoprotective molecules. The activated RSPCs would differentiate along a particular cell lineage and replace the damaged kidney cells. During acute kidney injury, the LRCs in the papilla migrated to the upper papilla and formed a compartment of rapidly proliferating cells, which may play a role in repair after ischemic injury [201]. The eventual descendants of LRCs differentiated into epithelial cells [202]. CD133^+^ cells are capable of differentiating into epithelial or endothelial cells in in vitro and in vivo [30, 203], and the renal homing and integration of in vitro expanded renal-derived CD133^+^ cells into some proximal and distal tubules of SCID mice with glycerol-induced acute tubulonecrosis were observed [30, 31, 204]. CD133^+^CD24^+^PDX^−^ cells when injected into mice with adriamycin-induced nephropathy glomerular, which are engrafted into the glomerular structures and tubular structures, accounted for 11.08 ± 3.3% of total podocytes and 7.5 ± 1.9% of all proximal tubular cells in mice. The results of intravenous injection of CD133^+^CD24^+^PDX^−^ cells reduced proteinuria and improved chronic glomerular damage of SCID mice suffering from nephrotic syndrome [193]. Shen et al. directed the differentiation of hiPSCs into endothelial progenitor cells (iEPCs). As expected, intravenously infused iEPCs were recruited to the injured kidney, replaced injured endothelial cells, and relieve kidney damage in AKI mice. Interestingly, iEPC therapy also ameliorated apoptosis of cardiomyocytes and cardiac dysfunction and no GFP-iEPCs in mouse hearts were observed. It was speculated that the protective effect of iEPC therapy on the heart may have resulted from the reduction of uremic toxin IS and proinflammatory IL-1β in circulation [205]. In addition, the injection of kidney SP cells into the AKI model demonstrated the recovery of renal function [198, 199]. However, some studies did not observe significant renal integration of SP cells and differentiation into renal cells was not observed as expected in some studies [199, 206].

A variety of cytokine/growth factors including IL-15, endothelial growth factor (EGF), and TNF-α that are secreted by RSPCs were detected, supporting a paracrine mechanism, similar to that described for MSCs might also contribute to systemic tissue repair [207, 208]. Bussolati et al. demonstrated that renal CD133^+^/CD73^+^ progenitors induce erythropoietin under hypoxia and inhibit prolyl pydroxylase [209]. Aggarwal et al. further provided evidence that the production and release of erythropoietin in in vivo stimulated by CD133^+^ renal progenitor cells contributed to the protection against fibrosis, limiting the presence of pro-inflammatory in SCID mice with AKI [189]. Recently, it has been reported that RSPCs protect physically injured or chemically damaged renal proximal tubular epithelial cells (RPTECs) by secreting regenerative molecules and microvesicles containing inhibin-A (Inhb-A) and decorin (DCN) [210]. Hishikawa and colleagues have reported that the SP cells isolated from the kidney with acute renal failure expressed high levels of renoprotective factors, such as HGF, VEGF, and leukemia inhibitory factor [198]. Also, the exosomes from conditioned medium of urine-derived stem cells (USCs-Exo) might have the potential to prevent kidney injury from diabetes by inhibiting podocyte apoptosis and promoting vascular regeneration and cell survival [194]. Since these cells could be conveniently obtained through non-invasive methods, urine-derived RSPCs possessed great potential in cell therapy and tissue regeneration. Furthermore, the release of pro-active renal protective factors from distal sites might also be involved, and this is because the intravenously injected RSPCs were localized in extrarenal organs such as the lungs and liver.

Despite the important role played in the repair and regeneration of damaged kidney tissue, a study showed that the dysregulated proliferation of renal progenitor cells generates hyperplastic glomerular lesions, scarring, and nephron loss eventually leading to degenerative disease such as crescentic nephritis or collapsing glomerulopathy [211].

Many researchers believe the presence of renal stem/progenitor cells, but a debate in this field is still going on and some different theories have been put forward. Kusaba et al. have showed that fully differentiated epithelial cells accomplish proximal tubule repair through reversible dedifferentiation and proliferation, without any contribution from the preexisting intratubular SC or progenitor population. In their study, injury to proximal epithelial tubule induced expression of markers of putative epithelial SCs in human kidney (CD24, CD133, and vimentin). What is more, no dilution of fate marker was observed when mice with completely labeled kidneys were subjected to injury and repair, indicating that unlabeled progenitors do not contribute to kidney repair [212]. Berger and colleagues have also suggested that there is no fixed progenitor cell population in the kidney, and tubular cells transiently acquire the phenotype of progenitor cells with reparative characteristics following injury [213].

## Challenges in the clinical translation of stem cells

Stem cells have shown great potential in kidney injury repair and disease treatment, but some pressing issues remain to be resolved before the clinical application. ESCs have limited clinical application due to ethical issues regarding their origin, tumorigenicity after transplantation. As the donor cells most likely do not originate from the recipient patient, immunological rejection of the cells differentiated from ESCs upon allogeneic cell transplantation remains a major challenge for cell therapy. Simultaneous administration of immunosuppressive drugs can aid in overcoming these problems but can induce serious side effects [214]. It has been proposed to overcome the immunogenicity of ESC-derived grafts by establishment of ESC donor banks covering various HLAs from selected homozygous HLA-typed volunteers. However, it is a labor-intensive, time-consuming work that is hard to carry out [215]. When it comes to iPSCs, the highly proliferative nature and the use of viral vectors for reprogramming exist with the risk of tumor development following transplantation, although some safer approaches such as the use of small molecules either have less reprogramming efficiency or usually cannot induce pluripotency alone. Reprogramming factors used to induce pluripotency (in particular, the proto-oncogenes c-MYC and KLF4) may become re-activated in the transplanted cells differentiated from iPSCs [216]. Moreover, frustrations due to low replication rate and premature aging were encountered in the administration of iPSCs.

Despite considerable body of evidence in in vitro supports the usefulness of MSCs, controversies about the use of SCs still persist. In clinical trials, the improvement of renal function and structure induced by MSCs in clinical trials is not as obvious as expected. The main reasons for the limited clinical efficacy were the low engraftment, the poor survival rate, and the impaired paracrine capacity of injected cells in vivo. The majority of grafted MSCs might be trapped in the lungs, liver, and spleen. For those cells engrafted into the target tissues, the harsh microenvironment still would induce apoptosis in over 90% of transplanted MSCs in 1 week. To improve the beneficial effects of MSC transplantation, the efficient strategy of administration of MSC needs to be discussed. A recent meta-analysis of MSC administration in kidney disease revealed that cell administration > 1 day after injury yielded greater therapeutic value than within 24 h of injury [217]. Liu et al. proposed that the timing of MSC administration should be immediately after ischemia to 1 h post-I/R. As the lack of inflammation in the early injured kidney was favorable for MSC survival within the tissue, favorable expression of homing adhesion molecules ICAM-1 and VCAM-1 were present, further promoting MSCs to integrate into injured kidney tissue [218]. In the mouse model of graft-versus-host disease, MSCs were most effective when administered at 3, 8, or 20 days after bone marrow transplantation [219]. In contrast, Casiraghi et al. proposed that injection of MSCs before transplantation rather than after it could achieve better immune suppression [176]. Therefore, the optimal timing of MSC administration for different kidney diseases remains under discussion. With more warnings, a long-term examination of intrarenal injection of MSCs in progressive rat model of glomerulonephritis has reported an abnormal and detrimental adipogenic differentiation of MSCs, suggesting the potential harm of administration of MSCs [218]. With regard to RSPCs, which showed the priority in displaying the inherent patient specificity at genomic level and kidney-specific epigenetic changes, the tissues from which autologous RSPCs can be obtained are poorly accessible or insufficient for cell isolation.

The immune rejection of stem cell injection is another challenge that hinders the clinical application of stem cell-based therapy. The allogeneic cells derived from hESCs will be robustly immune rejected by the recipient due to the direct activation of T cells by the foreign MHCs. While the patient-specific iPSCs share the same genetic background, and their derivatives might have a better chance to be immune tolerated, the genomic instability and abnormal epigenetics associated with iPSCs can induce immunogenicity in some cells of their derivatives [220]. MSCs have long been reported to be hypoimmunogenic or “immune privileged.” However, recent studies suggest it is not appropriate to consider MSCs to be immune privileged because they could elicit a humoral and cellular immune response in vivo. For example, whereas culture-expanded MSCs express low levels of MHC class me and are negative for MHC class II, this will likely be activated and/or expressed when MSCs are exposed to IFN-γ or differentiated into mature cell types [221]. Combining MSCs and anti-rejection drugs or a use modified MSC that reduces immunogenicity may be the possible strategies to overcome rejection of all MSCs [222].

## Conclusion

Whatever the sources or the approaches utilized such as ESCs, iPSCs, MSCs, or RSPCs, it is widely acceptable that there is an enormous hope underlying the usage of SC therapy as a clinically viable alternative for kidney diseases. Recent advances in cell-based therapy using various cell sources have demonstrated great promise towards restoring normal kidney functions in the AKI, DN, and CKD models, etc. The kidney organoids induced by stem cells provide the authentic and practical models for investigating kidney development and disease and progressing understanding about tissue regeneration, drug screening, and disease modeling. Although the stem cells from different sources show great promise as therapeutic agents for kidney diseases, the amount of research currently available is insufficient to achieve the docking of different kidney diseases with the most suitable transplanted stem cells. But interestingly, because of their immunomodulatory properties, MSCs may be the most promising candidates for stem cell therapy of autoimmune-related kidney diseases. In addition, MSCs obtained from autologous sources in sufficient amounts allow early treatment of patients affected by AKI. There is a need to carry out appropriately designed experimental studies to verify long-term safety of these therapies and to examine the risks of fibrosis, maldifferentiation or malignancy, and AEs of systemic immune suppression. Only the results of larger, well-powered rigorously designed clinical trials will assist in determining the real clinical efficacy of SC therapy in kidney diseases. Additionally, there are more factors that should be taken into consideration in the clinical application of SC therapy, such as appropriate selection of cell types, number of cells required, and appropriate route of administration. These combined efforts favor the translation of experimental results into clinical practice in the near future.

## Data Availability

All data generated or analyzed during this study are included in this article.

## Abbreviations

AKI: Acute kidney injury
AEs: Adverse events
ADPKD: Autosomal dominant polycystic kidney disease
AD-MSCs: Adipose-derived mesenchymal stem cells
ARPKD: Autosomal recessive polycystic kidney disease
BMPs: Bone morphogenic proteins
BM-MSCs: Bone marrow-derived mesenchymal stromal cells
CD44-HA: CD44-hyaluronic acid
CKD: Chronic kidney disease
CR: Complete remission
CXCR4: Chemokine (C-X-C motif) receptor 4
DN: Diabetic nephropathy
ESRD: End-stage renal disease
eGFR: Estimated glomerular filtration rate
ESCs: Embryonic stem cells
EVs: Extracellular vesicles
HGF: Hepatocyte growth factor
HSCs: Hematopoietic stem cells
hCB-MSCs: Human cord blood mesenchymal stem cells
hESCs: Human embryonic stem cells
hiPSCs: Human induced pluripotent cells
hPSCs: Human pluripotent stem cells
hUC-MSCs: Human umbilical cord-derived mesenchymal stem cells
iPSCs: Induced pluripotent cells
IM: Intermediate mesoderm
ISCs: Intestinal stem cells
LN: Lupus nephritis
LRCs: Label-retaining cells
MSCs: Mesenchymal stem cells
MSC-EVs: Mesenchymal stem cell-derived extracellular vesicles
MPCs: Mesenchymal precursor cells
MM: Metanephric mesenchyme
NSCs: Neural stem cells
PKD: Polycystic kidney disease
PSCs: Pluripotent stem cells
RFI: Renal failure induction
PR: Partial remission
RSPCs: Renal stem/progenitor cells
RVD: Renovascular disease
SCs: Stem cells
SAEs: Severe adverse events
SCID: Severe combined immunodeficient
TSCs: Totipotent stem cells
UB: Ureteric bud
VEGF: Vascular endothelial growth factor
ZGA or EGA: Activation of zygotic or embryonic genome
